# A Path Analysis Study on the Influence of Social Norms on Substance Use Severity: Focusing on People Who Use Cannabis, Narcotics, and Psychotropic Substances in South Korea

**DOI:** 10.3390/ijerph22010015

**Published:** 2024-12-27

**Authors:** Songhee Lee, Hyung-Ui Baik, Juyong Lee, Yunjae Shin

**Affiliations:** 1 Policy Research Department, Seoul Welfare Foundation, Seoul 04147, Republic of Korea; 2 Department of Addiction Rehabilitation and Social Work, Eulji University, Seongnam 13135, Republic of Korea; 3 Wholesome Day Counseling and Research Center, Seoul 08734, Republic of Korea; ljfromeast@gmail.com; 4 Suicide Action Forum, Seoul 07214, Republic of Korea; swsyj@naver.com

**Keywords:** drug, psychotropic substances, health beliefs, addiction severity

## Abstract

This study investigates the effects of social norms on substance use severity mediated by health beliefs among people who use cannabis, narcotics, and psychotropic substances in Republic of Korea. A survey was administered to 109 people who use cannabis and narcotics and 191 people who use psychotropic substances between May and July 2024. Path analysis was conducted. The findings indicated that the effects of social norms on health beliefs and the impact of health beliefs on substance use severity were statistically significant among people who use psychotropic substances, whereas no such significance was observed among people who use cannabis and narcotics. We recommend implementing early intervention treatment and social rehabilitation programs tailored to the specific needs and severity of substance use in people who use psychotropic substances, cannabis, and narcotics in Republic of Korea. Additionally, the establishment of online platforms to disseminate information on harm reduction, prevention, treatment, and rehabilitation based on substance type is advocated.

## 1. Introduction

In recent years, the prevalence of substance use Republic of Korea has escalated significantly, prompting the government to declare a ‘war on drugs’. This issue has emerged as a prominent social concern. According to the Ministry of Justice, the number of substance offenders in Republic of Korea increased from 9984 in 2014 to 18,395 last year [1,2]. Substance-related offenses are particularly prevalent among young individuals, with a 2020 survey by the Ministry of Food and Drug Safety indicating that individuals in their twenties demonstrated the lowest awareness of the seriousness of substance use. The illicit, excessive, and overprescribed use of medical substances has also led to significant adverse effects, with 1 in 2.7 individuals reporting the use of anesthetics, painkillers, and appetite suppressants.

Currently, the judicial response to people using substances in Republic of Korea consists of compulsory treatment, which includes therapeutic custody, correctional treatment, and probation. Conversely, conditional probation mandates participation in treatment and education, with non-compliance leading to prosecution, effectively making it compulsory. Correctional treatment allows participation in substance use disorder (SUD) programs only if the incarcerated individual agrees to treatment. Moreover, although individuals may voluntarily seek treatment and rehabilitation, the public substance use disorder (SUD) management system is largely ineffective due to the lack of community infrastructure to support recovery [1]. While some private organizations provide fragmented rehabilitation services, these are insufficient [2].

Social norms significantly influence individuals’ substance use and attitudes toward substance use disorder (SUD), shaped by factors such as popular culture, societal attitudes, and peer groups [3,4]. Griffiths et al. [5] highlight that digital platforms normalize substance use, lowering the perceived risks. In Eastern countries like China and Japan, social norms are more repressive. In China, substance use is seen as a severe crime, with individuals facing stigma and exclusion, leading to legal punishment rather than treatment [6,7]. Furthermore, the digital divide and cultural stigma in rural China further hinder treatment access for substance use disorder (SUD) [8]. Similarly, Japan has a strong stigma toward substance use, with individuals often facing exclusion and legal consequences [9,10]. In Republic of Korea, strong social norms and stigma surround substance use. Individuals with substance use disorder (SUD) are often seen as criminals, and the focus on legal punishment over treatment creates significant barriers. This societal perception may deter individuals from seeking help [11,12]. Furthermore, societal attitudes continue to emphasize the criminal nature of addiction, reinforcing punishment over rehabilitation [13]. Kim et al. [14] also note that legal penalties and stigma in in Republic of Korea hinder SUD treatment access.

Experiences from the United States and other countries indicate that investment in treatment, rehabilitation, and prevention programs is more effective than punitive measures [15,16]. The recent increase in substance use disorder (SUD) cases can be attributed to a lack of adequate treatment, rehabilitation, and prevention initiatives [3,4]. Although the need for substance use disorder (SUD) treatment and rehabilitation is increasingly recognized in Republic of Korea, discrepancies exist between the approaches of the Ministry of Justice, the Ministry of Food and Drug Safety, and the Ministry of Health and Welfare, particularly between punitive and therapeutic approaches to substance use disorder (SUD) [17,18,19]. This highlights the necessity for comprehensive research on substance use disorder (SUD) in Republic of South Korea; however, only a limited number of studies have been conducted thus far.

In Republic of Korea, substances are categorized into three classifications: narcotics, cannabis, and psychotropic substances, as defined by the Narcotics Control Act [18]. Due to its cultural characteristics, the use of narcotics is illegal in Republic of Korea and is subject to severe penalties. Drugs in Republic of Korea are classified into three categories: narcotics, psychotropic substances, and cannabis. First, narcotics include opium, morphine, heroin, cocaine, and fentanyl. The second category of psychotropic substances includes amphetamine, LSD, and ketamine. The third category is cannabis. Some drugs are used for medical purposes, but they must also be prescribed by a doctor. Since then, monitoring has been thoroughly conducted. In this way, in Republic of Korea, policies are being carried out with a strong focus on punishing substances, while alcohol is not subject to legal punishment in Republic of Korea. However, in the case of alcohol problems, various treatment and rehabilitation services are available through related hospitals and centers to help people solve their alcohol problems.

This research holds significant academic value as it is the first to differentiate between people who use cannabis, narcotics, and psychotropic substances in Republic of Korea, based on data from the Republic of Korea Ministry of Food and Drug Safety, and it encompasses a substantial survey sample.

Furthermore, this study examines the interrelations among social norms, health beliefs, and substance use severity among people who use cannabis, narcotics, and psychotropic substances Republic of Korea, to utilize the findings to develop prevention and rehabilitation programs tailored to each group.

The specific research objectives are:

(1) To identify the general characteristics, social norms, health beliefs, and severity of substance use among people of Republic of Korea who use cannabis, narcotics, and psychotropic substances
(2) To analyze the effects of social norms on substance use severity, mediated by health beliefs among people of Republic of Korea who use cannabis, narcotics, and psychotropic substances.

## 2. Materials and Methods

### 2.1. Study Design

Supported by the Ministry of Food and Drug Safety (MFDS) of Republic of Korea, this study examined the mediating effect of health beliefs on the relationship between social norms and substance use severity across two groups. Offline surveys were conducted among 109 people who use cannabis and 191 people who use psychotropic substances in Republic of Korea from May to July 2024. Path analysis was utilized to compare the effects of social norms on substance use severity, mediated by health beliefs, between people who use cannabis, narcotics, and psychotropic substances.

This study can be considered the first study to be conducted in the form of face-to-face interviews with drug addicts in Republic of Korea. To date, people who use substances in Republic of Korea are subject to severe punishment for illegal substance use, and the only way to find research subjects is to receive cooperation from prisons, the Ministry of Justice, and probation offices.

As mentioned earlier, the classification of drugs in Republic of Korea is divided into three categories: narcotics, psychotropic substances, and cannabis. First, substances include opium, morphine, heroin, cocaine, and fentanyl. The second category of psychoactive substances includes amphetamine, LSD, and ketamine. The third category is cannabis. The scope of the target population accessible under this classification system is limited, and the number is also small. Therefore, in this study, substance addicts who agreed to participate in this study through institutions that can cooperate with the investigation were classified into two groups. The first group is made up of people who use narcotics and cannabis, and the second group is made up of people who use psychotropic substances.

In this study, we intend to use the Health Belief Model, which was developed based on Lewin’s [20] field theory, as a theoretical model. The Health Belief Model is a model for predicting and analyzing health behavior, which views decisions about individual behavior as being made by the individual’s subjective perception rather than by the physical environment.

Since its development in the early 1950s, the Health Belief Model has been used in various public health issues. The Health Belief Model is a type of expectation-value model that assumes that a person will engage in a certain behavior when they have a perceived value and expectation that the behavior will prevent or alleviate disease [21]. In other words, the Health Belief Model is an expectation-value theory that sees a new behavior or change in behavior as occurring when an individual has the expectation that a particular behavior will prevent or alleviate disease and when the individual feels that it is worthwhile to prevent or alleviate a particular disease [22]. Accordingly, this study also sought to verify the mediating effect of health beliefs on drug addicts.

In this regard, previous studies have found that when positive cues to behavior are given in health beliefs, perceived threat, which consists of perceived severity and perceived vulnerability, increases, perceived benefits increase, and perceived barriers decrease. It is understood that desirable behavior and attitude factors increase as perceived threat increases and perceived barriers decrease [23,24]. Therefore, we will examine whether there is statistical significance in the variables related to health beliefs among the drug user population.

### 2.2. Study Population

This study targeted individuals who have used substances in Republic of Korea, collaborating with Korean probation offices, hospitals, Narcotics Anonymous, and other organizations. In Republic of Korea, substance use is illegal due to cultural characteristics, and there are strong penalties and regulations in place regarding the recruitment of research subjects. Even in the case of substances use for medical treatment purposes, it must be carried out according to a doctor’s prescription, and strong monitoring is being carried out.

Therefore, in order to recruit a sample of research subjects for this study, a snowball sampling method was conducted in cooperation with the Ministry of Justice, prisons, probation offices, and the Korea Anti-Substances Movement Headquarters. The survey was conducted only on those who agreed to participate in this study through the IRB. As such, there may be limitations in understanding and generalizing the characteristics of substance addicts in Republic of Korea due to the limitations of recruiting research subjects.

In addition, this study conducted a quantitative study to examine the characteristics of people who use substances. The minimum sample size for quantitative research varies depending on the number of estimated parameters, the magnitude of the effect, the power, and the estimation method. Jackson [25] proposes to calculate the sample size based on the ratio of the model’s estimated parameters and the sample size through the ‘N:q hypothesis’. In other words, Jackson’s proposal suggests that the ratio of the most ideal observation cases and the estimated parameters is 20:1, and the lowest level is 10:1.

Accordingly, the number of cases in this study was set to at least 300, with the ratio of the number of cases to the sample size set to the lowest level of 10:1, as suggested by Jackson [25], and the number of cases was set to 300. Jackson [25] suggests that the number of cases should be calculated based on the ratio of the number of cases to the sample size and the number of parameters in the model through the ‘N:q hypothesis’. In other words, Jackson’s proposal suggests that the ratio of the number of cases to the number of parameters is 20:1, which is the most ideal, and the lowest level is 10:1.

A total of 109 participants reported cannabis and marijuana use, while 191 reported use of psychotropic substances. Participants completed a self-administered questionnaire via online and offline surveys. The measurement instruments included the 15-item (5-point Likert scale) Social Norms Scale by Son Ae-ri et al. [19], the 9-item (5-point Likert scale) Health Belief Scale developed by Kim Jun-hong et al. [26], and the 10-item (dichotomous) Addiction Severity Scale by Skinner [27], as utilized by Kim [28]. As a result of verifying the reliability and validity of the scales used in this study, the reliability of each scale was high in existing prior studies as well. Similarly, the reliability of social norms was Cronbach’s a = 0.912, the reliability of substance use severity was Cronbach’s a = 0.833, and the reliability of health beliefs was Cronbach’s a = 0.847.

### 2.3. Ethical Considerations

The survey of people who use cannabis, narcotics, and psychotropic substances in Republic of Korea was conducted following ethical approval from the Institutional Review Board of Eulji University. Informed consent was obtained from all participants prior to data collection, and this study was reviewed by the Institutional Review Board of the research institution (IRB No. EUIRB2024-048).

### 2.4. Data Analysis

**Figure 1:**
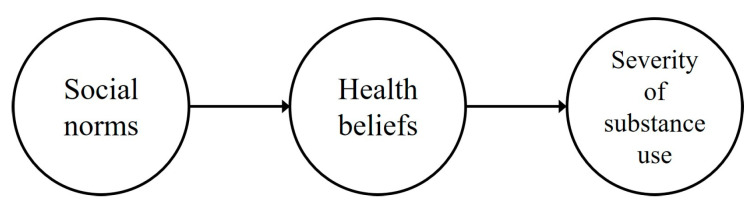
Research model (path of social norms to the severity of substance use mediated by health beliefs).

Data analyses were conducted using IBM SPSS (Statistical Package for the Social Sciences) Statistics for Windows v. 27.0 (IBM Corporation, Armonk, NY, USA) and AMOS (Analysis of Moment Structures) 21.0. Descriptive statistics and correlation analyses were performed using SPSS, while path analysis was conducted using AMOS. Descriptive statistics characterized the demographic, social norms, health beliefs, and substance use severity among people who use cannabis, narcotics, and psychotropic substances in the sample. Correlations were evaluated among these variables, and path analyses were performed to assess the influence of social norms and health beliefs on substance use severity, separating the two user groups as in Figure 1.

#### Main Variables

First, this is related to the general characteristics of the research participants. Participants’ gender was categorized as ‘male’ or ‘female’; educational attainment ranged from ‘primary school’ to ‘graduate or higher’; and marital status included ‘single’, ‘married/cohabiting’, ‘separated’, ‘divorced’, ‘widowed’, and ‘other’. Age was classified from ‘teenager’ to ‘60s’.

Second, the Social Norms Scale comprised items relating to perceptions of substance use, encompassing a total of 15 items associated with addiction stigma, personal stigma, social stigma, social norms, and tolerant attitudes. Each item was rated on a 5-point scale from ‘strongly disagree’ to ‘strongly agree’.

Third, the Health Beliefs Scale consisted of nine items that evaluated attitudes towards substance use, including perceptions of susceptibility and severity, barriers, and benefits. Each item was rated on a 5-point scale from ‘not at all’ to ‘strongly agree’.

Finally, this is the part related to the severity of substance use. The Addiction Severity Scale comprised 10 items, each requiring a dichotomous ‘yes’ or ‘no’ response regarding experiences related to substance use in the past year.

## 3. Results

### 3.1. General Characteristics of the Participants

This study included a total of 300 people who use substances, comprising 109 people who use cannabis and narcotics and 191 people who use psychotropic substances. The gender distribution consisted of 218 (72.9%) males and 81 (21.7%) females. The age distribution included 2 (0.7%) teenagers, 113 (38.4%) in their 20s, 104 (35.4%) in their 30s, 48 (16.3%) in their 40s, 21 (7.1%) in their 50s, and 6 (2.0%) aged 60 or older. Educational attainment was as follows: 1 (0.3%) elementary school graduate, 3 (1.0%) junior high school graduates, 25 (8.4%) high school graduates, 152 (50.8%) vocational college graduates, 67 (22.4%) university graduates, and 13 (4.3%) with a graduate degree or higher. Marital status included 215 (72.1%) single, 52 (17.4%) married/cohabiting, 3 (1.0%) separated, 25 (8.4%) divorced, 1 (0.3%) widowed, and 2 (0.7%) classified as other.

### 3.2. Descriptive Statistics and Correlation of Two Groups: People Who Use Cannabis and Narcotics and People Who Use Psychotropic Substances

Table 1 presents the correlation and descriptive statistics of social norms, health beliefs, and substance use severity. Among people who use cannabis and narcotics, the correlations between social norms and health beliefs (r = −0.043, *p* > 0.05) and substance use severity (r = 0.185, *p* > 0.05), as well as between health beliefs and substance use severity (r = 0.054, *p* > 0.05), were statistically insignificant. Conversely, for people who use psychotropic substances, there were positive correlations between social norms and health beliefs (r = 0.593, *p* < 0.01) and health beliefs and substance use severity (r = 0.473, *p* < 0.01). However, the correlation between social norms and substance use severity was not significant (r = 0.087, *p* > 0.05). In addition, the normality assumption of the measured variables was checked, and the intercept values of people who use cannabis and narcotics’ reasoning were within 3 (0.47–2.04), and the skewness values (0.33~9.04) were within 10; the skewness values (0.00~51) of people who use psychotropic substances were within 1, and the skewness values (1.06~3.20) were within 4. Each variable used in this study was found to be within the range of normal distribution, confirming that the assumption of normality was met for each measured variable.

### 3.3. The Impact of Social Norms and Health Belief Characteristics on Substance Use Severity

#### 3.3.1. Goodness of Fit

The results of the goodness-of-fit test of the research model are shown in Table 2. The fit of the research model was significant (*p* < 0.001) with a χ^2^ value of 4.341, df = 2, and the fit was determined to be generally acceptable with Normed (χ^2^/df) = 2.170, TLI = 0.835, CFI = 0.945, NFI = 0.911, IFI = 0.950, and RMSEA = 0.063 (0.000~0.145).

#### 3.3.2. Results of the Path Analysis of Social Norms and Health Beliefs to Health Beliefs and Severity of Substance Use

**Figure 2:**
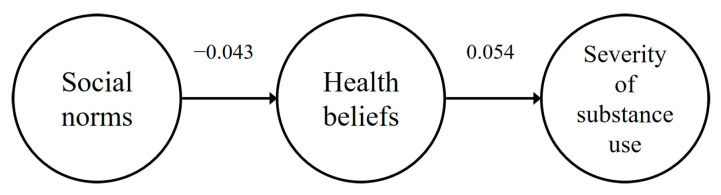
Path of the research model (people who use cannabis and narcotics).

**Figure 3:**
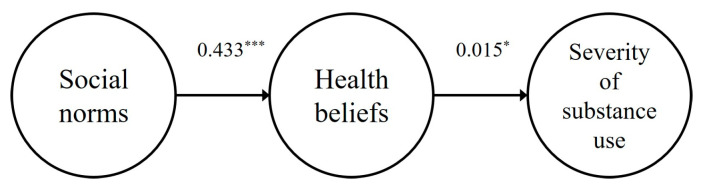
Path of the research model (people who use psychotropic substances). *** *p* < 0.001, * *p* < 0.05.

The results of the causal analysis of the paths in the research model are shown in Table 3 and Figure 2 and Figure 3. The direct paths from social norms to health beliefs (β = −0.043, *p* > 0.05) and health beliefs to substance use severity (β = 0.054, *p* > 0.05) were not statistically significant for people who use cannabis and narcotics, but the direct paths from social norms to health beliefs (β = 0.433, *p* < 0.001) and health beliefs to substance use severity (β = 0.150, *p* < 0.05) were positively significant for people who use psychotropic substances.

### 3.4. The Direct and Indirect Effect Decomposition

To investigate the effect of exogenous variables of the research model analyzed in this study on endogenous variables, effect decomposition was conducted. The results of the effect decomposition to verify the direct and indirect effects of the research model are shown in Table 4. The results of the direct and indirect effect decomposition of the research model show that there are two direct pathways and one indirect pathway. First, the direct paths of social norms → health beliefs (β = −0.043, *p* > 0.05) and health beliefs → substance use severity (β = 0.054, *p* > 0.05) were not statistically significant for people using cannabis and narcotics, and the indirect path of social norms → substance use severity (β = −0.002, *p* > 0.05) was not statistically significant. For people who use psychotropic substances, the direct paths from social norms to health beliefs (β = 0.433, *p* < 0.001) and health beliefs to substance use severity (β = 0.150, *p* < 0.05) were positively significant, and the indirect path from social norms to substance use severity (β = 0.065, *p* < 0.05) was positively significant.

This means that while strong social norms, such as those of medicinal users, can have a significant impact on reducing the severity of substance use, the strong educational effects associated with social norms may not have a significant impact on people’s use of cannabis and narcotics. Therefore, there should be a difference in the approach to treatment and rehabilitation education for each type of substance.

## 4. Discussion

This study aimed to determine the effect of social norms on substance use severity through health beliefs among two groups of people: those who use cannabis and narcotics, and those who use psychotropic substances in Republic of Korea from May to July 2024. This study was conducted as part of a study by the Ministry of Food and Drug Safety (MFDS) in 2024. The results of the analysis showed that the effects of social norms on health beliefs and health beliefs on substance use severity were statistically significant among people who use psychotropic substances, and the effects of social norms on substance use severity were also statistically significant. Consistent with previous studies, we found evidence that social norms among people influence health-related attitudes [1,8,29,30,31]. However, some recent studies have shown that social norms also influence substance use severity [32]. As depression, lack of self-control, stress, and low self-esteem have been suggested as relapse factors in substance use disorder (SUD), it is of academic interest that social norms and health beliefs have also been shown to influence substance use severity in this study [19,26,30,31,33]

However, for people who use cannabis and narcotics, the effects of social norms on health beliefs and health beliefs on substance use severity were not statistically significant, and the indirect path from social norms to substance use severity was not statistically significant. These results suggest that prevention education and social rehabilitation programs for people who use substances in Korea need to take into account the type of substances; i.e., strong social norms may have a significant effect on reducing substance use severity in some cases, such as people who use psychotropic substances, but strong education effects related to social norms may not have a significant effect in the case of people who use cannabis and narcotics. Furthermore, the recent surge in substance problems in Republic of Korea has been centered on young people [17,34,35], and it is therefore necessary to develop a substance-specific and age-specific approach in the future. It is necessary to provide education to prevent substance use for each type of substance and to positively cope with substance risks.

## 5. Conclusions

While the substance problem in Republic of Korea is overgrowing, substance offenses in Asia, such as Singapore, Japan, and Thailand, have long been associated with high recidivism rates. Also, as shown in many Asian countries, it is difficult to reduce the recidivism rate by emphasizing punishment alone, like other crimes [2,18,27,29,30]. Currently, there are many limitations to the current policies to prevent recidivism and relapse among people with substance use disorder (SUD) in Republic of Korea [21,35,36]. This is because Republic of Korea’s substance policy still emphasizes regulation and punishment. Previous studies have also pointed out that Republic of Korean substance policy tries to reduce the substance problem through criminal punishment without understanding substance use disorder (SUD). For this reason, they point out that the high recidivism rate of substance offenses and the punishment-oriented policy without appropriate early intervention for substance problems further exacerbate the problem [27,30].

In recent years, the Korean Mental Health and Social Welfare Association, the Ministry of Food and Drug Safety, and others have begun to train professionals for the treatment and rehabilitation of substance use disorder (SUD). However, currently there is a severe shortage of treatment and rehabilitation centers and professional staff for substance use disorder (SUD) [18,37,38]. Furthermore, there is a lack of institutions and service programs that can differentiate between treatment and education for people who use cannabis and people who use psychotropic substances.

There is also a need for a system to link the justice system of Republic of Korea with the substance treatment and rehabilitation system. In particular, various programs should be set up to prevent the escalation of the substance problem by age group and type of drug; i.e., intensive preventive education should be provided to high-risk youth and adults identified through probation centers of Republic of Korea, mental health centers, and integrated substance use disorder (SUD) management support centers. In addition, a system should be in place to provide early social rehabilitation services to those who have used cannabis, narcotics, and psychotropic substances and to refer them to case management services. In addition, various online platforms should be established to provide information on harm, prevention, treatment, and rehabilitation for different types of substances; i.e., online websites should provide information on organizations where people can receive treatment for substance use disorder (SUD) to increase access to treatment and rehabilitation services.

In conclusion, this study highlights the pressing need for the development of targeted intervention strategies that consider the unique characteristics of people who use psychotropic substances in Republic of Korea. The establishment of comprehensive online platforms that provide extensive resources on harm reduction, prevention, treatment, and rehabilitation is strongly recommended. Such platforms could serve as vital tools in disseminating evidence-based information and facilitating greater awareness of the health risks associated with substance use.

In particular, in the future, it is necessary to focus on developing, educating, and guiding programs for early prevention of substance use disorder (SUD) and return to school in the health and welfare sector, especially in centers that support adolescents or young adults. The insights gleaned from this research have significant implications for public health initiatives aimed at addressing substance use disorder (SUD) in Republic of South Korea. By acknowledging and addressing the roles of social norms and health beliefs, stakeholders can enhance the effectiveness of intervention programs and contribute to the broader goal of reducing substance use severity across various user groups.

Future studies should seek to expand on these findings by investigating additional variables that may influence substance use severity, as well as employing longitudinal designs to capture the evolving dynamics of substance use and recovery over time. By doing so, researchers can contribute to a more comprehensive understanding of substance use disorder (SUD) and inform policy decisions that foster healthier communities.

Finally, as mentioned in the introduction, the use of cannabis is illegal in Republic of Korea and has been enforced with a strong policy of punishment. The social norms are also different from those of other countries where the use of cannabis is legal, which means that the approach to drug addicts should be different for those who have lived abroad (in countries or regions where drugs are legal) and those who have not. Moreover, the people of Republic of Korea’s awareness of the seriousness of drug problems is becoming stronger. However, in recent years, the number of teenagers and those in their twenties who approach these drug problems simply out of curiosity or for recreational purposes is increasing, as social norms are not important. Therefore, it is suggested that follow-up studies should be actively conducted on the differences between approaches to drug use in overseas cases and literature and the limitations of drug use approaches in the Korean context.

## Figures and Tables

**Table 1:** Descriptive statistics and correlation. (A) People who use cannabis and narcotics (n = 109). (B) People who use psychotropic substances (n = 191).

Variables	1. Social Norms		2. Health Beliefs		3. Severity of Substance Use	
1. Social norms	1		−0.043		0.185	
2. Health beliefs	0.433 **		1		0.054	
3. Severity of substance use	0.109		0.150 *		1	
**Variables**	**A**	**B**	**A**	**B**	**A**	**B**
Mean	3.43	3.35	3.40	3.52	4.08	4.58
SD	0.47	0.55	0.62	0.69	2.50	2.89
Skewness	0.73	−0.35	−2.04	−0.51	9.04	0.00
Kurtosis	1.25	3.20	9.04	1.26	−0.33	−1.06

Notes: Results for people who use cannabis and narcotics are above the diagonal, and people who use psychotropic substances are below the diagonal. ** *p* < 0.01, * *p* < 0.05.

**Table 2:** Goodness-of-fit measures for the path analysis.

Measure	χ^2^	df	Normed	TLI	CFI	NFI	IFI	RMSEA	
								LO90	HI90
Comparison model	4.341	2	2.170	0.835	0.945	0.911	0.950	0.063	
								0.000	0.145

**Table 3:** Path analysis of the research model. (A) People who use cannabis and narcotics (n = 109). (B) People who use psychotropic (n = 191).

Path	Estimate		S.E.	C.R.	*p*
	*B*	*β*			
(A) Social norms → Health beliefs	−0.057	−0.043	0.128	−0.445	0.656
(A) Health beliefs → Severity of substance use	0.219	0.054	0.385	0.568	0.570
(B) Social norms → Health beliefs	0.540	0.433	0.082	6.612 ***	0.0009
(B) Health beliefs → Severity of substance use	0.630	0.150	0.301	2.093 *	0.036

*** *p* < 0.001, * *p* < 0.05.

**Table 4:** Decomposition of total effects into direct and indirect effects. (A) People who use cannabis and narcotics (n = 109). (B) People who use psychotropic substances (n = 191).

Path	Estimate (*β*)		
	Direct Effect	Indirect Effect	Total Effect
(A) Social norms → Health beliefs	−0.043	-	−0.043
(A) Social norms → Severity of substance use	-	−0.002	−0.002
(A) Health beliefs → Severity of substance use	0.054	-	0.054
(B) Social norms → Health beliefs	0.433 ***	-	0.433 ***
(B) Social norms → Severity of substance use	-	0.065 *	0.065 *
(C) Health beliefs → Severity of substance use	0.150 *	-	0.150 *

*** *p* < 0.001, * *p* < 0.05.

## Data Availability

The data supporting this study are available upon request from the Ministry of Food and Drug Safety (MFDS) of the Republic of Korea.
